# Type 2 sclerotic Modic change affect fusion result in patients undergoing PLIF with pedicle screw instrumentation: a retrospective study

**DOI:** 10.1186/s12891-021-04461-9

**Published:** 2021-06-28

**Authors:** Hao Li, Shou Chen, He-Yu Wei, Chuang-Ye Han, Fan-Yue Zeng, Shuang-Shuang Yuan, Hong-Yu Qin, Jin-Song Yang

**Affiliations:** 1 Guangxi Medical University, 22 Shuangyong Road, Nanning, Guangxi China; 2 The Fourth Affiliated Hospital of Guangxi Medical University, 1 Liushi Road, Liuzhou, Guangxi China; 3 Department of Orthopedics, The First Hospital of Fangshan District, 6 Fangyao Road, Beijing, China; 4 Department of Orthopedics, The First Affiliated Hospital of Guangxi Medical University, 22 Shuangyong Road Nanning, Nanning, 530022 Guangxi China

**Keywords:** Modic changes, Endplate sclerosis, Posterior lumbar interbody fusion, Pedicle screw instrumentation, Bony fusion

## Abstract

**Background:**

Bony fusion rate was significantly lower in patients with type 3 Modic change than patients with normal endplates. It is not known whether there are relevant differences in fusion efficiency among patients with type 2 sclerotic Modic change or non-sclerotic Modic change, or no Modic change.

**Methods:**

A retrospective study contained 196 lumbar segments in 123 subjects undergoing posterior lumbar interbody fusion (PLIF) with pedicle screw instrumentation (PSI) to assess the effect of type 2 sclerotic Modic change on fusion efficiency. These endplates were allocated into groups A, B, and C, according to their Modic changes. Group A had endplates with type 2 Modic change and endplate sclerosis. Group B had type 2 Modic change without endplate sclerosis. Group C had neither Modic change nor endplate sclerosis. The presence of Modic change was determined by magnetic resonance imaging (MRI). Endplate sclerosis in type 2 Modic change was detected by computed tomography (CT) before the operation. We collected CT data 3 months to more than 24 months after operation in patients to assess bony fusion.

**Results:**

Incidences of bony fusion were 58.8% in group A, 95.0% in group B, 94.3% in group C. The bony fusion rate was significantly lower in group A than in either group B or C. There was no significant difference between groups B and C. Thus, endplates with type 2 sclerotic Modic change had a lower fusion rate in patients undergoing PLIF with PSI.

**Conclusion:**

Type 2 sclerotic Modic change could be an important factor that affects solid bony fusion in patients undergoing PLIF with PSI. CT may help diagnose endplate sclerosis in patients with type 2 change and inform the choice of the best site for spinal fusion.

## Background

Vertebral endplate (Modic) changes are abnormalities of the endplate and adjacent bone marrow that can be seen with magnetic resonance imaging (MRI). A classification of these changes was first provided by Modic et al. (Modic et al., 198,801) based on the evaluation of 474 patients in 1988. Type 1 change was defined as a hypointense signal on T1-weighted images and a hyperintense signal on T2-weighted images. Type 2 change was defined as a hyperintense signal on T1-weighted images and isointense or slightly hyperintense signals on T2-weighted images, reflecting the fatty replacement of the bone marrow. Type 3 change was a hypointense signal on T1- and T2-weighted images.

In lumbar fusion surgery, there was a strong negative correlation between type 3 Modic change and bone graft fusion. Besides, there was no correlation between type 1 or 2 Modic change and bone graft fusion. In a study involving 351 patients who underwent PLIF with threaded fusion cages (TFC), Kwon et al. [1], showed the bony fusion rate after PLIF was lower in patients with Modic change than in those without Modic change, only in patients with type 3 Modic change. Endplate sclerosis is the pathological feature of the Modic changes type 3, just because of endplate sclerosis, the fusion rate in patients with Modic changes type 3 is lower than type 2 and type 1. Generally, endplate sclerosis exists only in type 3 Modic changes, but not in type 1 and 2 [2–4]. However, recent studies revealed that sclerosis can occur in endplates with any type of Modic changes, especially in type 2 [5–7].

Mari et al. [7] reported a total of 82 Modic changes at 216 endplates (38%). Of these changes, 53 (65%) were type 2, and one (1%) type 3. Twelve (22.6%) endplates with Modic changes type 2 in MRI had sclerosis in CT. In clinical practice, we have noticed that type 3 Modic changes are extremely rare among patients, and endplate sclerosis is more frequently observed in patients with Modic changes type 2 using computed tomography (CT) images. Based on the above background, the number of Modic changes in type 2 is much more than that in type 3, and the endplate sclerosis is more frequently observed with type 2 Modic change when comparing with type 1. Therefore, exploring the effect of type 2 sclerotic Modic change on bone graft fusion is significant and urgent.

However, the effect of type 2 sclerotic Modic change on fusion efficiency in patients undergoing PLIF with pedicle screw instrumentation (PSI) is unclear. The purpose of the current study was to assess the effect of type 2 sclerotic Modic change on fusion efficiency in patients undergoing PLIF with PSI.

## Methods

### Study participants

This was a retrospective study. The study was conducted at a single institution between January 2009 and March 2018, and consisted of 123 patients (56 men, 67 women) who underwent PLIF with PSI. A total of 196 lumbar segments recorded in 123 subjects were allocated into groups A, B, and C according to the endplate changes: 1. Group A had endplates with type 2 Modic change and endplate sclerosis. 2. Group B had type 2 Modic change but without endplate sclerosis. 3. Group C had neither Modic changes nor endplate sclerosis. According to fusion potentiality at L5-S1 level would be lower than the upper lumbar levels [8], segments of the three groups were further divided into two subgroups: L5-S1 segment (groups A1, B1, C1) and L1–5 segments (groups A2, B2, C2). After reviewing the digital database of a radiology record system, patients meeting the following criteria were included: (1) patients age was more than 18 years; (2) patients who had been diagnosed with lumbar spondylolisthesis or lumbar spinal canal stenosis; (3) patients underwent lumbar spine surgery with pedicle screw instrumentation, and the decompressed space was implanted with cage; (4) patients had no history of adolescent scoliosis, spinal surgery, tumor, tuberculosis, infection and trauma; (5) patients did not smoke before surgery and had no smoking during the postoperative follow-up; (6) patients were not diagnosed with osteoporosis (*T* score ≤ − 2.5) or had very low osteoporosis risk (female < 55 years, male < 60 years); (7) patients were not diagnosed with hypertension, diabetes and heart disease. For the main purpose is to discuss the difference in fusion efficiency among patients with type 2 sclerotic Modic change or non-sclerotic Modic change, a few patients with type 3 Modic change were excluded.

### Ethics statement

The research was conducted according to the principles of the Declaration of Helsinki. The ethics committee of the First Affiliated Hospital of Guangxi Medical University approved the study and written informed consent was obtained from all patients (2019(KY-E-033)).

### Operative technique

Patients were operated on in a prone position under general anesthesia. A midline incision was made to expose the spinous processes, laminae, and transverse processes. The initial stage involved inserting posterior transpedicular screw instrumentation (Common Spinal Fixation Device, Ltd., Li Bell, China) through a paraspinal muscle-splitting approach. The transpedicular screws were inserted under C-arm fluoroscopic guidance in all patients. The next stage involved posterior decompression (including laminectomy, medial facetectomy, and amniotomy), which was undertaken in all patients. A nearly complete discectomy was done. Intervertebral disc space spreaders were then inserted sequentially and rotated to restore the normal disc space. Next, an appropriate size of the cage was inserted into the disc space directly under C-arm fluoroscopy so it would lay in the middle of the interbody space.

### Imaging analysis

**Fig. 1:**
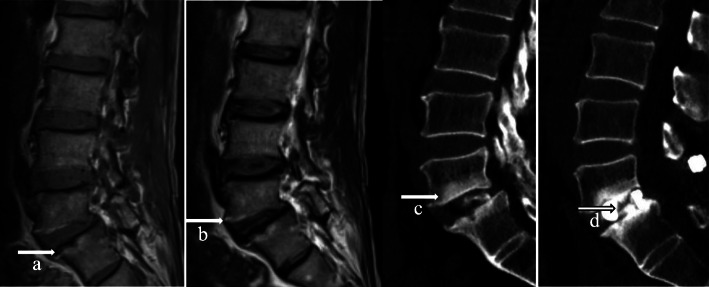
50–59-year-old patient with type 2 sclerotic Modic change (indicated by arrow)—high T1- and T2-weight magnetic resonance imaging (MRI) signals—showing the anatomical areas. a, b Sagittal T1-weighted (**a**) and T2-weighted (**b**) MRI scans show high signal at the L5-S1 endplates. Sagittal reconstructed (**c**) computed tomography (CT) scans show sclerosis at the L5-S1 endplates. Sagittal reconstructed (**c**, **d**) CT scans at the 12-month follow-up evaluationshow that there was no evidence of trabecular bone formation extending from the upper or lower endplate of L5-S1 (no fusion)

Modic changes were determined using MRI (GE Signa Twinspeed; GE Medical Systems, Milwaukee, WI, USA), and endplate sclerosis was detected on pre-operative sagittal and coronal reconstructed CT scans (GE Light Speed Pro 16; GE Healthcare, Milwaukee, WI, USA). MRI and CT analyses included the operated lumbar levels. Endplate sclerosis was seen adjacent to the endplate and usually localized in the same area as the lumbar interbody fusion Modic change (Fig. 1). At 3 months or longer after surgery, the patients were evaluated with CT. Classification of Modic changes was based on the T1- and T2-weighted MRI results in the middle five sagittal planes. The upper and lower endplates at each disc level were graded separately regarding the presence of type 2 Modic change or absence of Modic change, as previously defined [2] (Fig. 1a,b). Endplate sclerosis was visually evaluated from the sagittal and coronal reconstructed CT scans by comparing them with the MRI at a workstation (Fig. 1a–d). The presence of endplate sclerosis was defined as yes or no. Bony fusion was evaluated according to the postoperative sagittal and coronal reconstructed CT scans (Fig. 1c, d). CT became the preferred method for assessing interbody fusion [9–13]. Details of the bony fusion evaluation were as follows [13–15]: (1) complete fusion: evidence of bridging trabecular bone through the disc space with no cystic lucencies adjacent to the implant and no linear defects through the bridging bone; (2) partial fusion: trabecular bone seen extending from the endplate into the disc space but forming an incomplete bridge; (3) no fusion: no evidence of trabecular bone formation extending from the endplates. Because this study aimed to assess the bony fusion of vertebral body endplates, both complete and partial fusion were considered fusion.

Three experienced spine surgeons (JL, FZ, and CZ.) who were blinded to the radiographic images independently classified the endplate changes and evaluated the images for the presence of bony fusion. If at least two of the observers agreed about the type of endplate change and the inter-observer agreement ICC over 0.75, the classification was carried out [16]. The binary logistic regression analysis was used to examine the association between type 2 sclerotic Modic change and bony fusion; three binary logistic regression analysis models were inputted in turn. The models were performed as follows: a model adjusted for groups A and C (model 1); a model adjusted for groups A and B (model 2); a model adjusted for groups B and C (model 3).

## Results

### Patient demographics

Patients (*N* = 123) were assigned with group A (type 2 sclerotic Modic change), group B (type 2 Modic change) or group C (no Modic change). Of 123 patients, 19 (15.4%) were group A, 21 (17.1%) were group B and 83 (67.5%) were group C. Demographic and baseline characteristics for three groups are presented in Table 1. Three groups were comparable regarding age (group A vs. B, *p* = 0.74, group A vs. C, *p* = 0.78, group B vs. C, *p* = 0.47), female gender (group A vs. B, *p* = 0.23, group A vs. C, *p* = 0.86, group B vs. C, *p* = 0.27), BMI (group A vs. B, *p* = 0.14, group A vs. C, *p* = 0.18, group B vs. C, *p* = 0.61), Lumbar spondylolistesis (group A vs. B, *p* = 0.58, group A vs. C, *p* = 0.85, group B vs. C, *p* = 0.60), single level fusion (group A vs. B, *p* = 0.91, group A vs. C, *p* = 0.11, group B vs. C, *p* = 0.13), and available DXA result (group A vs. B, *p* = 0.14, group A vs. C, *p* = 0.57, group B vs. C, *p* = 0.18).

**Table 1:** Demographic and baseline characteristics of the patients

	Group A	Group B	Group C	*p*-value^a^	*p*-value^b^	*p*-value^c^
No. of patients, n (%)	19 (15.4)	21 (17.1)	83 (67.5)	NA	NA	NA
Age (years), mean (SD)	53.0 (9.74)	54 (9.80)	52.2 (10.26)	0.74	0.78	0.47
Female, n (%)	10 (52.6)	14 (66.7)	43 (51.8)	0.23	0.86	0.27
Body mass index (kg/m^2^), mean (SD)	23.3 (1.81)	22.3 (2.06)	22.5 (2.14)	0.14	0.18	0.61
Preoperative *T* score, (n available)	−1.64 (7)	−1.23 (8)	− 1.51 (27)	0.14	0.57	0.18
No. of segments, n (%)	34 (17.4)	40 (20.4)	122 (62.2)	NA	NA	NA
Spondylolisthesis, n (%)	8 (42.1)	7 (33.3)	33 (39.8)	0.58	0.85	0.60
Spinal canal stenosis, n (%)	11 (57.9)	14 (66.7)	50 (60.2)	0.58	0.85	0.60
Single level fusion, n (%)	6 (31.6)	7 (33.3%)	43 (51.8)	0.91	0.11	0.13
Multiple level fusion, n (%)	13 (68.4)	14 (66.7%)	40 (48.2)	0.91	0.11	0.13
Follow-up period (CT imaging), mean 12.0 (3–39)						

All values are expressed as the mean (standard deviation = SD), except proportions (%)

Nonparametric tests were performed for data that failed the normal distribution

Group A type 2 sclerotic Modic change, Group B type 2 Modic change, Group C no Modic change

^a^Group A vs. B, ^b^Group A vs. C, ^c^Group B vs. C

### Modic change and sclerosis in endplates

Among a total of 196 endplates from 123 patients, 74 (37.8%) had evidence of type 2 Modic change. Of these 196 segments, 34 (17.4%) exhibited type 2 sclerotic change (group A), 40 (20.4%) had type 2 nonsclerotic change (group B), and 122 (62.2%) had no Modic change (group C) (Table 2).

**Table 2:** Number of segments in three groups at different levels(*N* = 196)

	Group			
Level	Group A	Group B	Group C	Total
L1–2			2	2 (1.0%)
L2–3			3	3 (1.5%)
L3–4	7	5	28	40 (20.4%)
L4–5	11	17	60	88 (44.9%)
L5-S1	16	18	29	63 (32.1%)
Total	34 (17.4%)	40 (20.4%)	122 (62.2%)	196

Group A type 2 sclerotic Modic change, Group B type 2 Modic change, Group C no Modic change

### Bony fusion in the three groups

**Fig. 2:**
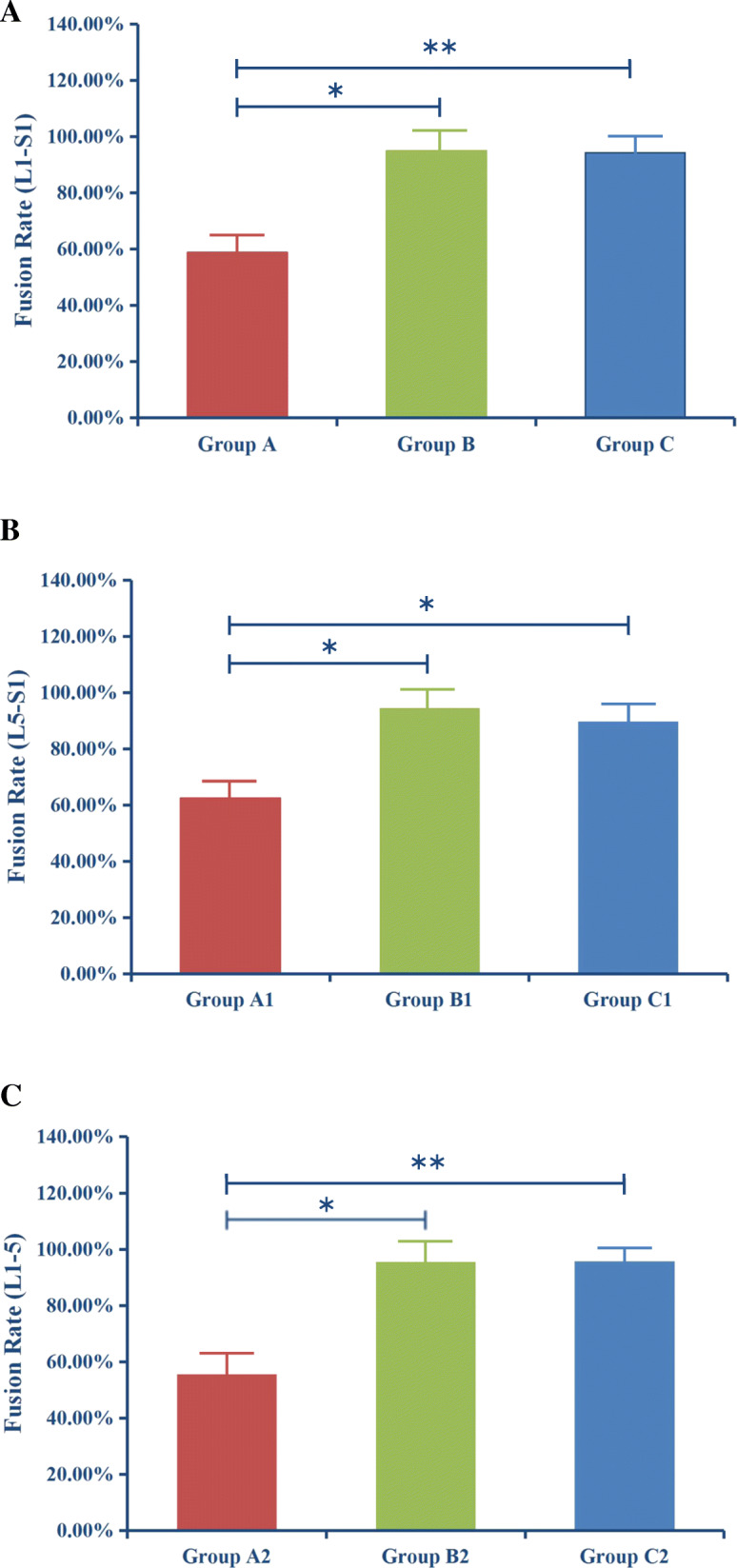
Fusion rates for the groups and subgroups. The fusion rates were 58.8% in group A, 95.0% in group B, and 94.3% in group C (**a**). In the subgroup (L5-S1), the bony fusion rates were 62.5% in group A1, 94.4% in group B1, and 89.7% in group C1 (**b**). In the subgroup (L1–5), the bony fusion rates were 55.6% in group A2, 95.5% in group B2, and 95.7% in group C2 (**c**). Group A type 2 sclerotic Modic change, Group B type 2 Modic change, Group C no Modic change. **Significantly low fusion rate (*P <* 0.01), *Low fusion rate (*P <* 0.05)

The bony fusion rates were 58.8% in group A, 95.0% in group B, and 94.3% in group C. The fusion rate was significantly lower in group A than in the other two groups (*P* < 0. 001), while there was no statistical difference in the fusion rates between groups B and C (*P =* 0. 860) (Fig. 2a) (Table 3).

**Table 3:** Independent predictors of bony fusion in patients undergoing PLIF with PSI (L1-S1)

Logistic Regression Models	β	Wald	*P* value	Odds ratio	95% CI
**Model 1**	**2.588**	**10.338**	**< 0.001**	**13.300**	**2.747–64.405**
**Model 2**	**2.442**	**21.852**	**< 0.001**	**11.500**	**4.130–32.021**
Model 3	−0.145	0.031	=0.860	0.865	0.172–4.342

Sample size, *n* = 196. Data are expressed as odds ratios ±95% confidence intervals (CI) as assessed by binary logistic regression analysis

Group A type 2 sclerotic Modic change, Group B type 2 Modic change, Group C no Modic change

All covariates included in binary regression models were as follows: model 1: groups A and C, model 2: groups A and B, model 3: groups B and C

### Bony fusion in the subgroups

In the subgroup, the bony fusion rates were 62.5% in group A1, 94.4% in group B1, and 89.7% in group C1. The fusion rate was lower in group A1 than in the other two groups (P < 0. 05), while there was no statistical difference in the fusion rates between groups B1 and C1 (*P* = 0. 573) (Table 4). The bony fusion rates were 55.6% in group A2, 95.5% in group B2, and 95.7% in group C2. The fusion rate was lower in group A2 than in the other two groups (P < 0. 05), while there was no statistical difference in the fusion rates between groups B2 and C2 (*P* = 0. 960) (Fig. 2b,c) (Table 5).

**Table 4:** Independent predictors of bony fusion in patients undergoing PLIF with PSI (L5-S1)

Logistic Regression Models	β	Wald	*P* value	Odds ratio	95% CI
**Model 1a**	**2.322**	**4.069**	**< 0.05**	**10.200**	**1.068–97.406**
**Model 2a**	**1.659**	**4.257**	**< 0.05**	**5.200**	**1.086–24.897**
Model 3a	−0.674	0.317	=0.573	0.510	0.049–5.315

Sample size, *n* = 63. Data are expressed as odds ratios ±95% confidence intervals (CI) as assessed by binary logistic regression analysis

Group A type 2 sclerotic Modic change, Group B type 2 Modic change, Group C no Modic change

All covariates included in binary regression models were as follows: model 1a: groups A1 and C1, model 2a: groups A1 and B1, model 3a: groups B1 and C1

**Table 5:** Independent predictors of bony fusion in patients undergoing PLIF with PSI (L1–5)

Logistic Regression Models	β	Wald	*P* value	Odds ratio	95% CI
**Model 1b**	**2.821**	**6.255**	**< 0.05**	**16.800**	**1.841–153.304**
**Model 2b**	**2.879**	**17.049**	**< 0.01**	**17.800**	**4.538–69.818**
Model 3b	0.058	0.003	=0.960	1.060	0.113–9.975

Sample size, *n* = 133. Data are expressed as odds ratios ±95% confidence intervals (CI) as assessed by binary logistic regression analysis

Group A type 2 sclerotic Modic change, Group B type 2 Modic change, Group C no Modic change

All covariates included in binary regression models were as follows: model 1b: groups A2 and C2, model 2b: groups A2 and B2, model 3b: groups B2 and C2

## Discussion

This study aimed to investigate the effect of bony fusion after PLIF with PSI in patients with type 2 sclerotic Modic change. In endplates with either lumbar spondylolisthesis or lumbar spinal canal stenosis, we found that bony fusion was positively associated with type 2 sclerotic Modic change. The bony fusion rate was 55.3% in group A, which is significantly lower than that in either group B or group C. However, there was no clear association between type 2 nonsclerotic Modic change and no Modic change in the fusion rate.

Interestingly, bony fusion rates in this study are somewhat different from those published previously. Earlier studies compared bony fusion rates of endplates with different types of Modic change and found that the bony fusion rates were lower than that of normal endplates [1, 17]. A study involving 351 patients who underwent PLIF with TFC had been conducted by Kwon et al., whose results showed that the bony fusion rate in each group of Modic change was as follows: 81% in type 1, 84% in type 2, 55% in type 3, and 97% in patients with no Modic degeneration. The bony fusion rate was significantly low in the patients with type 3 Modic change [1]. One reason for the difference might be the existence of endplate sclerosis in type 2 change, but those authors did not further classify type 2 sclerotic Modic change into subgroups.

The bony fusion rate of endplates with type 2 sclerotic Modic change is significantly lower than that in patients with no Modic change after PLIF with PSI—a point worthy of preoperative attention.

Regarding the pathology of Modic change, it has been reported that type 2 change showed bone marrow being replaced with abundant fat [2, 18]. Type 2 change showed high signal intensity on T1-weighted images and isointense or slightly hyperintense signal on T2-weighted images. Shaikh et al. [19, 20] reported that low-signal-intensity reactive sclerosis was observed on both T1- and T2-weighted images. Due to low-signal-intensity reactive sclerosis was covered by high-signal-intensity reactive fat. In type 2 sclerotic Modic change, endplate sclerosis might not be seen on MRI. Preoperative CT examinations of every patient with Modic change would be wise.

It has been suggested that endplate sclerosis exists in different Modic types, especially in type 3 change and mixed Modic change, which (mixed Modic change) means that inflammation (type 1) and fatty (type 2), fatty and sclerotic (type 3) or inflammation and sclerotic are simultaneous existence in the same endplate [21], and that it can be detected by CT [7]. Endplate sclerosis in type 3 Modic change was a reflection of densely mineralized bone in the vertebral body rather than the marrow elements [2]. Kuisma et al. believed that sclerosis seen in most of the mixed Modic types and some types 1 and 2 change might reflect a regenerative process in the marrow with new bone formation. Hence, they speculated that reactive sclerosis seen in Modic change on CT scans might reflect a healing process of the bone marrow [7]. In our study, however, the bony fusion rate was lower in the presence of type 2 sclerotic change than in the presence of type 2 nonsclerotic change or no Modic change. Therefore, we speculated that endplate sclerosis in type 2 change—which was similar to that seen in type 3 Modic change via plain radiography or CT—was a reflection of densely mineralized bone in the vertebral body rather than completely a regenerative process with new bone formation. Thus, endplate sclerosis in type 2 Modic change may reduce blood supply to the vertebral body–graft interface, leading to fusion delay or failure. This assumption needs to undergo more studies that involve histopathological evidence.

**Fig. 3:**
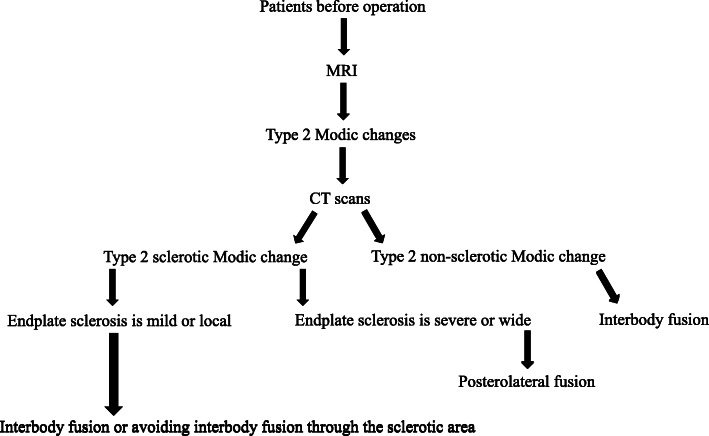
Simplified algorithm for imaging patients with type 2 Modic change

As a result of the above analysis, we propose a simple algorithm for imaging patients with type 2 Modic change. If the patients’ MRI scan shows type 2 Modic change, CT should be performed as a routine examination before surgery. It might also provide a definitive imaging basis for the most advantageous location for spinal fusion. If the endplate sclerosis is mild or local, we would have a choice of interbody fusion or avoiding interbody fusion through the sclerotic area. If the endplate sclerosis is severe and widespread, our choice would be posterolateral fusion (Fig. 3). The limitation of this research was that the sample size was too small. Since this is a retrospective study, we judged the smoking status only by individual medical record. But the medical record did not include whether they had a history of smoking, how many cigarettes they smoke every day, whether they had given up smoking, etc. And only a portion of patients did exam the dEXA (dual energy X-ray absorptiometry) before operation. To further understand the influence of these factors on bone fusion rate, further research is needed.

## Conclusion

Our results support the possibility that type 2 sclerotic Modic change could be an important factor that affects solid bony fusion in patients undergoing PLIF with PSI. We should pay more attention preoperatively to patients with type 2 sclerotic Modic change, including the performance of a preoperative MRI/CT examination and determine the best site for spinal fusion.

## Data Availability

The data that support the findings of this study are available from the First Affiliated Hospital of Guangxi Medical University. but restrictions apply to the availability of these data, which were used under license for the current study, and so are not publicly available. Data are however available from the authors upon reasonable request and with permission of the First Affiliated Hospital of Guangxi Medical University..

## Abbreviations

PLIF: Posterior lumbar interbody fusion
PSI: Pedicle screw instrumentation
MRI: Magnetic resonance imaging
CT: Computed tomography
TFC: Threaded fusion cages

## References

[1] Kwon YM, Chin DK, Jin BH, Kim KS, Cho YE, Kuh SU (2009). Long term efficacy of posterior lumbar interbody fusion with standard cages alone in lumbar disc diseases combined with Modic changes. Journal of Korean Neurosurgical Society.

[2] Modic MT, Steinberg PM, Ross JS, Masaryk TJ, Carter JR (1988). Degenerative disk disease: assessment of changes in vertebral body marrow with MR imaging. Radiology.

[3] Modic MT, Masaryk TJ, Ross JS, Carter JR (1988). Imaging of degenerative disk disease. Radiology.

[4] Wu HL, Ding WY, Shen Y, Zhang YZ, Guo JK, Sun YP, Cao LZ (2012). Prevalence of vertebral endplate modic changes in degenerative lumbar scoliosis and its associated factors analysis. Spine.

[5] Chi S, Hongli W, Jianyuan J, Feizhou L, Xiaosheng M, Xinlei X (2018). The pathology of type II Modic changes: fat deposition or Osteosclerosis? A study using CT scan. Biomed Res Int.

[6] Liu J, Huang B, Hao L, Shan Z, Zhang X, Chen J, Fan S, Zhao F (2019). Association between Modic changes and endplate sclerosis: evidence from a clinical radiology study and a rabbit model. J Orthop Transl.

[7] Kuisma M, Karppinen J, Haapea M, Lammentausta E, Niinimaki J, Tervonen O (2009). Modic changes in vertebral endplates: a comparison of MR imaging and multislice CT. Skelet Radiol.

[8] Aono H, Takenaka S, Nagamoto Y, Tobimatsu H, Yamashita T, Furuya M, Iwasaki M (2018). Fusion rate and clinical outcomes in two-level posterior lumbar interbody fusion. World Neurosurg.

[9] Burkus JK, Dorchak JD, Sanders DL (2003). Radiographic assessment of interbody fusion using recombinant human bone morphogenetic protein type 2. Spine.

[10] Chafetz N, Cann CE, Morris JM, Steinbach LS, Goldberg HI, Ax L (1987). Pseudarthrosis following lumbar fusion: detection by direct coronal CT scanning. Radiology.

[11] Cook SD, Patron LP, Christakis PM, Bailey KJ, Banta C, Glazer PA (2004). Comparison of methods for determining the presence and extent of anterior lumbar interbody fusion. Spine.

[12] Rothman SL, Glenn WV. CT evaluation of interbody fusion. Clin Orthop Relat Res. 1985:47–56. 10.1097/00003086-198503000-00006. 3971636

[13] Williams AL, Gornet MF, Burkus JK (2005). CT evaluation of lumbar interbody fusion: current concepts. AJNR Am J Neuroradiol.

[14] Shah RR, Mohammed S, Saifuddin A, Taylor BA (2003). Comparison of plain radiographs with CT scan to evaluate interbody fusion following the use of titanium interbody cages and transpedicular instrumentation. Eur Spine J.

[15] Huang W-m, Yu X-m, Xu X-D, Song R-X, Yu L-L (2017). Xiu-Chun Yu (2017) posterior lumbar interbody fusion with Interspinous fastener provides comparable clinical outcome and fusion rate to pedicle screws. Orthop Surg.

[16] Jensen TS, Sorensen JS, Kjaer P (2007). Intra- and interobserver reproducibility of vertebral endplate signal (modic) changes in the lumbar spine: the Nordic Modic consensus group classification. Acta Radiol.

[17] Chataigner H, Onimus M, Polette A (1998). Surgery for degenerative lumbar disc disease. Should the black disc be grafted?. Revue de chirurgie orthopedique et reparatrice de l'appareil moteur.

[18] Toyone T, Takahashi K, Kitahara H, Yamagata M, Murakami M, Moriya H (1994). Vertebral bone-marrow changes in degenerative lumbar disc disease. An MRI study of 74 patients with low back pain. J Bone Joint Surg Br.

[19] Shaikh MI, Saifuddin A, Pringle J, Natali C, Sherazi Z (1999). Spinal osteoblastoma: CT and MR imaging with pathological correlation. Skelet Radiol.

[20] Tobias A, Azeem A, Alisson R, Jean C, Dzung H, Todd D (2017). The 'Lumbar fusion outcome Score' (LUFOS): a new practical and surgically oriented grading system for preoperative prediction of surgical outcomes after lumbar spinal fusion in patients with degenerative disc disease and refractory chronic axial low back pain. Neurosurg Rev.

[21] Dudli S, Fields AJ, Samartzis D, Karppinen J, Lotz JC (2016). Pathobiology of Modic changes. Eur Spine J.

